# Eosinophil cytolysis with or without ETosis in four cases of human gastric cancer: a comparative ultrastructural study

**DOI:** 10.37349/etat.2025.1002309

**Published:** 2025-04-21

**Authors:** Rosario Caruso, Valerio Caruso, Luciana Rigoli

**Affiliations:** IRCCS Istituto Romagnolo per lo Studio dei Tumori (IRST) “Dino Amadori”, Italy; ^1^Department of Human Pathology in Adult and Developmental Age “Gaetano Barresi”, University of Messina, 98123 Messina, Italy; ^2^Clinical and Experimental Medicine Department, University of Pisa, 56100 Pisa, Italy

**Keywords:** Gastric carcinoma, eosinophil, eosinophil extracellular traps, eosinophil cytolysis, ultrastructure

## Abstract

The ultrastructural morphology of eosinophil cytolysis and extracellular trap cell death (ETosis) has predominantly been examined in non-neoplastic eosinophil-associated diseases, with a limited investigation in neoplasms. This current electron microscopy study examined the ultrastructural characteristics of eosinophil cytolysis and ETosis across four distinct gastric cancer cases: three cases (cases 1–3) exhibited non-ETotic cytolysis, while one case (case 4) presented eosinophils at various stages of ETosis. In cases 1–3, eosinophil non-ETotic cytolysis was characterized by localized plasma membrane disruption, the presence of free extracellular granules (FEGs), and the maintenance of a round or oval nuclear lobe profile. In case 4, eosinophils were observed in progressive stages of ETosis, arbitrarily subdivided into early, intermediate, and advanced. Although early ETosis and non-ETotic cytolysis exhibited overlapping ultrastructural features, chromatin decondensation and nuclear envelope enlargement were more pronounced in early ETosis. Nuclear envelope disruption, loss of the round or oval nuclear lobe profile (intermediate stage), extracellular DNA trap deposition, and the appearance of Charcot-Leyden crystals (advanced stage) were all distinctive features of ETosis. The findings of this case report confirm previous observations of eosinophil cytolysis with or without ETosis in non-neoplastic diseases and extend them to advanced gastric carcinoma. Since Charcot-Leyden crystals were only seen in case 4, their correlation with ETosis was further supported. In gastric cancer, the release of FEGs during non-ETotic cytolysis and the release of both FEGs and DNA traps during ETotic cytolysis may contribute to the formation of an antitumor microenvironment.

## Introduction

Eosinophils are a type of innate immune cell that have a significant impact on several disorders, such as asthma, helminth parasite infections, and cancer [1]. A recent meta-analysis demonstrated that tumor-associated tissue eosinophilia (TATE) is a favorable prognostic indicator, especially in patients with gastrointestinal cancer [2]. Electron microscopy has shown that eosinophil degranulation occurs through distinct mechanisms [3]. These mechanisms include classical exocytosis, where individual granules fuse with the plasma membrane and release their full contents; compound exocytosis, where intracellular granules fuse with each other before releasing their contents into the extracellular space; piecemeal degranulation, where eosinophilic sombrero vesicles transport small packets of materials from granules to the cell surface; and cytolysis, where cells release intact granules [3]. In 2013, Ueki et al. [4] identified a subtype of eosinophil cytolysis that is associated with extracellular trap cell death (ETosis). This process involves the extracellular release of both specific granules and nuclear chromatin [4], as also demonstrated in non-neoplastic eosinophil-associated diseases [5–11]. Recently, we provided ultrastructural evidence of eosinophil ETosis in a case of poorly cohesive carcinoma, non-signet-ring cell type (PCC-NOS) of the stomach [12]. In this study, we conducted a retrospective review of our institutional experience with gastric cancers characterized by TATE. The objective was to evaluate the ultrastructural characteristics of eosinophil cytolysis in three additional cases of gastric cancer. Therefore, we evaluated eosinophil cytolysis with and without ETosis to compare and identify their ultrastructural characteristics in the tumor stroma of gastric cancer.

## Case report

### Materials and methods

At the Department of Human Pathology in Adult and Developmental Age “Gaetano Barresi”, University of Messina, Italy, gastric tumors were systematically prepared for light and electron microscopic analysis. The fresh tumor tissue fragments were divided into two portions using a sharp razor blade. The initial specimen of the pair underwent standard paraffin embedding procedures. The second sample of the paired specimens was minced into smaller fragments, which were promptly fixed in 3% phosphate buffered glutaraldehyde at pH 7.4, postfixed in 1% osmium tetroxide, dehydrated using graded ethanols, and subsequently embedded in Araldite. Ten blocks were prepared, and semithin sections were stained with Toluidine blue to allow field selection. Ultrathin sections were double-stained with uranyl acetate and lead citrate for electron microscopy. They were then examined using a Zeiss EM 902 electron microscope (Carl Zeiss, Oberkochen, Germany) and a JEOL 1200 electron microscope (JEOL, Tokyo, Japan).

### Results

We selected four cases of gastric carcinomas in which eosinophil cytolysis and ETosis were documented by electron microscopy. Table 1 presents a summary of the pertinent clinicopathological findings.

**Table 1:** Clinicopathological characteristics of gastric cancer with TATE

**Case number**	**Age (years)**	**Sex**	**Location**	**Size (cm)**	**pTNM stage**	**Histological type**
1	66	Male	Antrum	4	T2N0M0	Tubular, moderately differentiated
2	74	Female	Body	3	T2N0M0	PCC-NOS
3	76	Male	Antrum	7	T2N2M0	Tubular, moderately differentiated
4	80	Female	Body	4	T2N1M0	PCC-NOS

TATE: tumor-associated tissue eosinophilia; PCC-NOS: poorly cohesive carcinoma, non-signet-ring cell type

**Figure 1:**
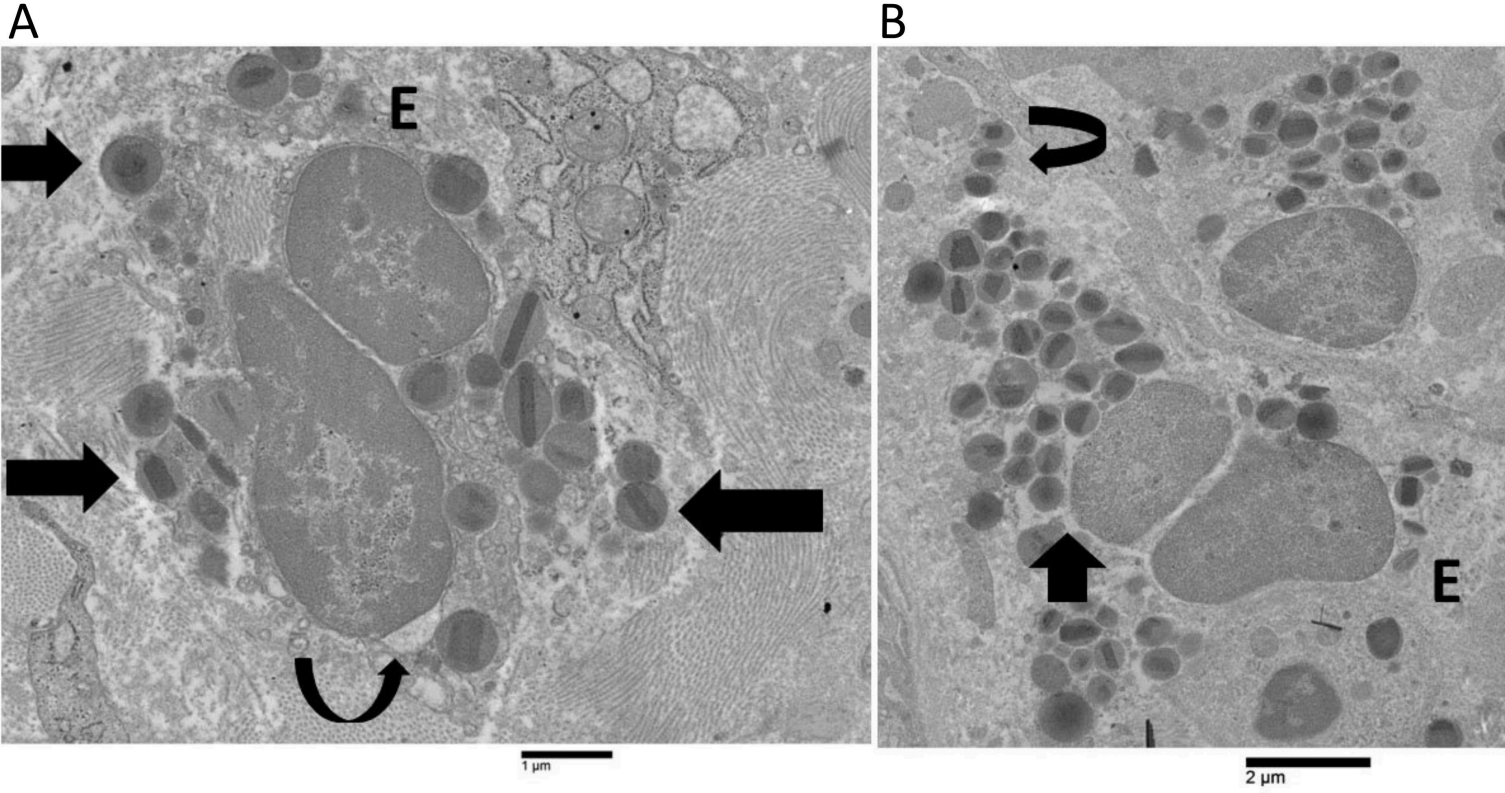
**Ultrastructural images of human eosinophils showing non-ETotic cytolysis and early extracellular trap cell death (ETosis)**. **A**) A non-ETotic cytolytic eosinophil (E) in patient 1 exhibits a localized expansion of the nuclear envelope (curved arrow), moderately decondensed chromatin, and free extracellular granules (FEGs) (arrows); **B**) the early ETotic eosinophil (E) in patient 4 shows a notable dilatation of the perinuclear space (arrow), extensively decondensed chromatin, and FEGs (curved arrow)

The electron microscopy analysis of patients 1, 2, and 3 detected a small number of eosinophils that exhibited non-ETotic cytolysis. This was characterized by incomplete heterochromatin decondensation, focal loss of plasma membrane, and the presence of free extracellular granules (FEGs) (Figure 1A). Nuclear membranes began to separate from one another forming irregular dilatation (Figure 1A), while nuclear lobes exhibited a round or oval morphology (Figure 1A). Decondensed chromatin was not spilled intracellularly or extracellularly. These patients did not exhibit Charcot-Leyden crystals. In patient 4, eosinophils showed ETotic changes that could be arbitrarily subdivided into early, intermediate, and advanced phases. These eosinophils showed a higher frequency of early and intermediate stages than advanced ETotic eosinophils. The early ETotic eosinophils had nuclear lobes that were rounded or oval-shaped (Figure 1B), with extensively decondensed and expanded chromatin (Figure 1B). Additionally, there was a focal loss of plasma membrane and the presence of FEGs (Figure 1B). The nuclear envelope exhibited an enlarged perinuclear space, as shown in Figure 1B.

**Figure 2:**
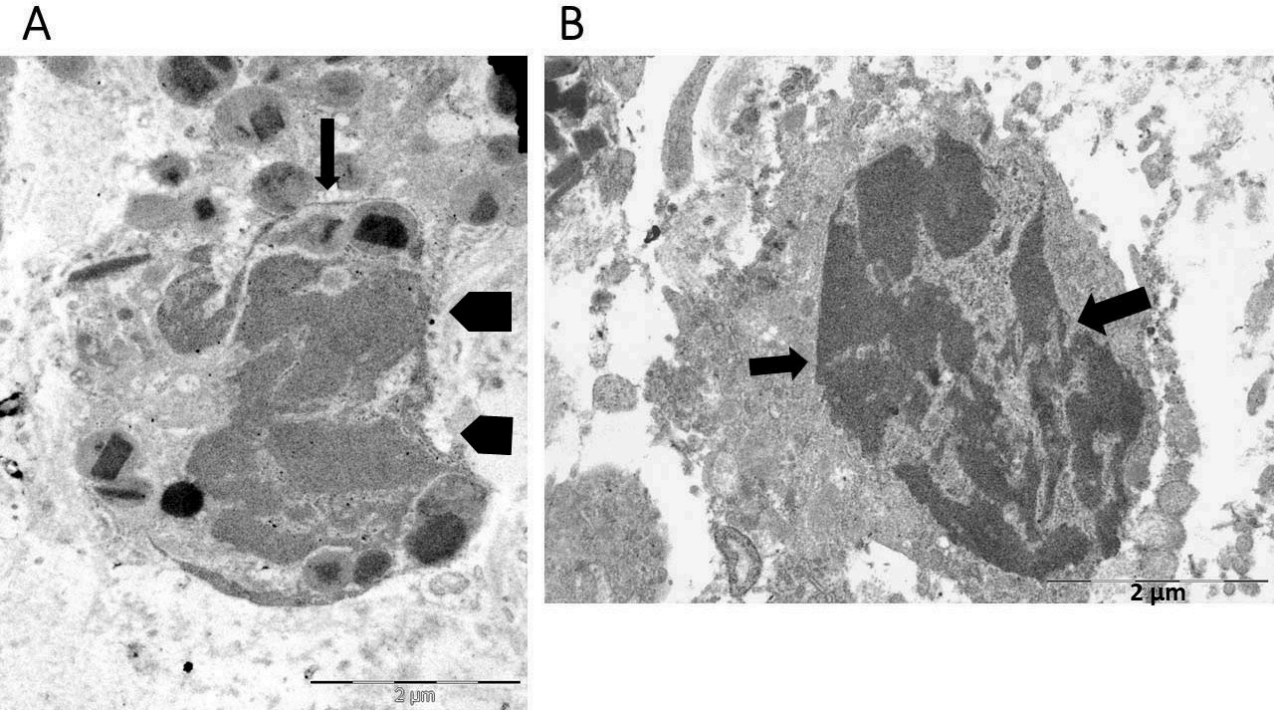
**Ultrastructural findings in the intermediate stage of eosinophil extracellular trap cell death (ETosis)**. **A**) Decondensed chromatin appears as serpiginous deposits (arrow) or irregular blocks (pentagonal arrows); **B**) this eosinophil has undergone complete degranulation and exhibits intracellular localization of decondensed chromatin blocks, connected by thin chromatin bridges (arrows)

**Figure 3:**
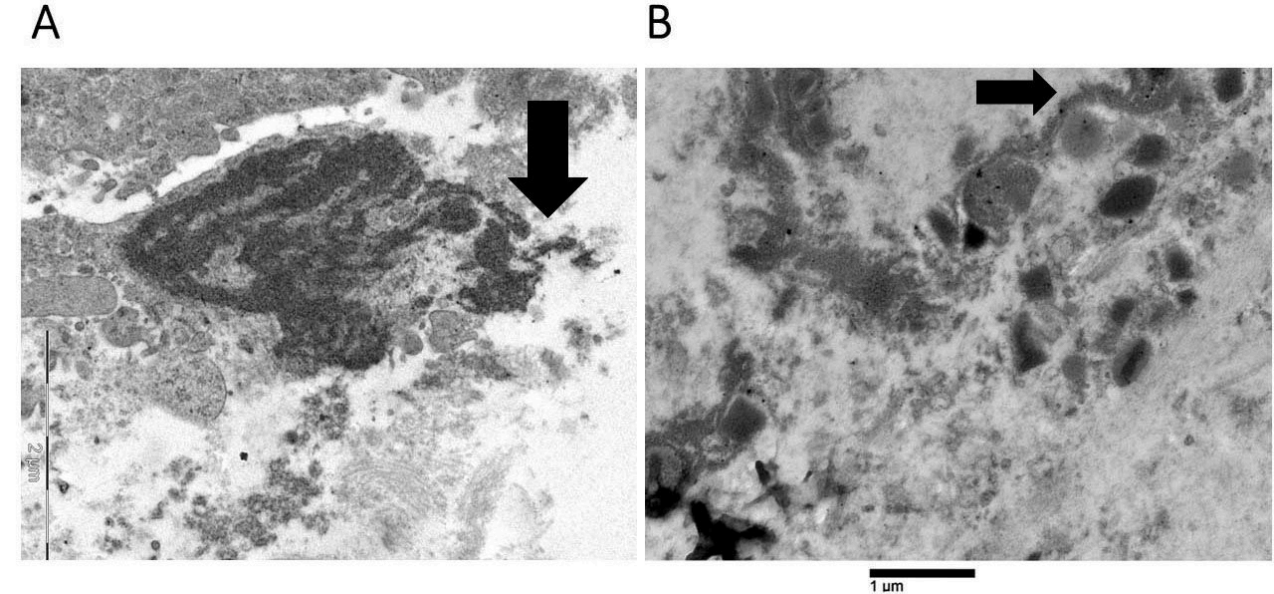
**Advanced stage of eosinophil extracellular trap cell death (ETosis)**. Extracellular DNA traps can be observed either as decondensed chromatin structures with irregular borders (arrow, as shown in **A**) or as serpiginous fibers in contact with free extracellular granules (FEGs) (arrow, as shown in **B**). The two electron microscopes used to acquire the images produced distinct scale bars. Each scale bar precisely denotes the magnification employed in the corresponding image

Eosinophil nuclear lobes lost their distinctive oval or round form during the intermediate stage of ETosis (Figure 2). This event was associated with the disintegration of the nuclear envelope and the subsequent release of decondensed chromatin into the cytoplasm (Figure 2). Decondensed chromatin was observed as either single blocks with irregular borders (Figure 2) or elongated serpiginous chromatin structures (Figure 2A). Delicate chromatin bridges connecting chromatin blocks were also visible (Figure 2B). The cytoplasm either lacked specific granules (Figure 2B) or only contained a small number (Figure 2A). The defining characteristics in advanced ETotic eosinophils were the extensive disintegration of the plasma membrane and the deposition of extracellular DNA traps. The traps were identified as either decondensed chromatin aggregates with irregular edges (Figure 3A) or serpiginous fibers that entangled FEGs (Figure 3B).

**Figure 4:**
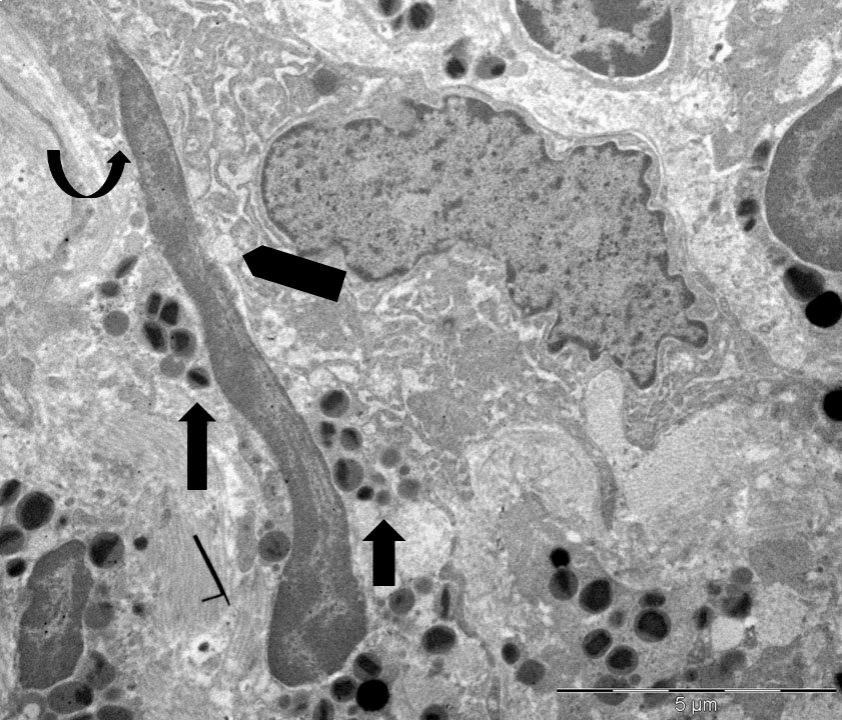
**Ultrastructure of extracellular DNA trap in the tumor microenvironment**. A spindle-shaped extracellular deposit of decondensed chromatin, measuring approximately 12 µm in length, is wedged in the cytoplasm of a single tumor cell (curved arrow). This shows focal loss of plasma membrane and dilation of the endoplasmic reticulum (pentagonal arrow). Free extracellular granules (FEGs) are also observed (arrows)

Figure 4 illustrates an uncommon spindle-shaped DNA trap wedged in the cytoplasm of a tumor cell, which reveals a localized loss of plasma membrane integrity and a dilatation of the rough endoplasmic cisternae.

**Figure 5:**
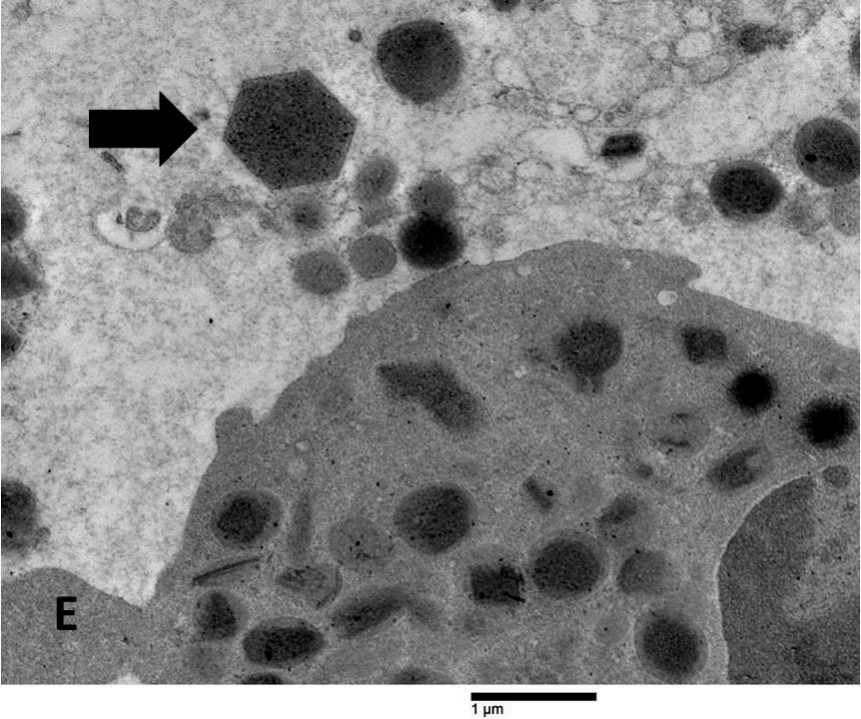
**Ultrastructural features of Charcot-Leyden crystals in the tumor stroma**. A hexagonal Charcot-Leyden crystal (arrow) can be seen beside an eosinophil (E) in an edematous extracellular region

Crystals displaying pyramidal, hexagonal, and asymmetrical forms, similar to Charcot-Leyden crystals, were seen in areas with FEGs (Figure 5).

## Discussion

In patient 4, our data show numerous eosinophils undergoing early, intermediate, and advanced ETosis. The ultrastructural characteristics of the early ETotic eosinophils in this case were similar to those of the cytolytic non-ETotic eosinophils observed in cases 1, 2, and 3. The observed characteristics consisted of focal loss of plasma membrane integrity, oval/round nuclear lobes profiles, FEGs, and incomplete decondensation of chromatin. In case 4, however, the process of chromatin decondensation and enlargement of the nuclear envelope was more pronounced in the early ETotic stage than in cytolytic non-ETotic eosinophils. Electron microscopy enables us to distinguish between early ETosis and non-ETotic cytolysis, despite the fact that they share some features. Intermediate and advanced ETosis are characterized by unique ultrastructural features. The intermediate phase of ETosis is distinguished by the disruption of the nuclear envelope, loss of round/ovoid nuclear lobes profiles, and the presence of irregular blocks of decondensed chromatin in the cytoplasm. Conversely, advanced ETosis is marked by the complete disruption of the plasma membrane, in addition to the extracellular deposition of Charcot-Leyden crystals and DNA traps. Our ultrastructural findings are similar to those reported previously in non-neoplastic eosinophil-associated diseases [5–11]. Consequently, they confirm and extend their findings to the microenvironment of stomach cancer.

In patient 4, eosinophils exhibiting ETosis were identified at various stages, with early and intermediate stages being more prevalent than advanced ETotic eosinophils. However, as this is a descriptive ultrastructural study based on a limited number of cases, we were not able to perform a quantitative assessment of the proportion of eosinophils in each stage. This represents a limitation of our study, underscoring the necessity for additional studies on larger cohorts to more accurately delineate the dynamics and prevalence of each ETosis stage in eosinophil-associated tumor microenvironments.

Charcot-Leyden crystals are composed of a single protein called galectin-10 [13]. They have been occasionally observed in cases of allergy and parasitic illnesses, acute myeloid leukemia, chronic eosinophilic leukemia, and papillary cancer of the pancreas [14]. Eosinophil ETosis is strongly associated with the production of Charcot-Leyden crystals [15, 16]. Their unique occurrence only in patient 4, characterized by the presence of numerous ETotic eosinophils, coincides with the observations documented in the literature.

FEGs, which are released following cytolysis/ETosis, function as autonomous active organelles that can initiate an active secretory response [17, 18]. There are highly cytotoxic preformed proteins in eosinophil specific granules. These include major basic proteins, eosinophil cationic proteins, eosinophil peroxidase, and eosinophil-derived neurotoxin [19]. Basic proteins, which are selectively released following cytolysis/ETosis, exhibit both immunoregulatory and cytotoxic properties in the tumor microenvironment [20–23]. Cationic DNA-binding histones, found in DNA traps, induce cell death in host cells through their interaction with negatively charged phospholipids in cell membranes. This interaction results in pore formation, increased permeability, and ultimately leads to cell lysis [24–26]. Within the framework of our investigation, eosinophils contribute to the tumor microenvironment by releasing extracellular traps, cytokines, and granules. These components influence tumor progression through modulation of immune responses, induction of cytotoxic effects, and alteration of the local inflammatory environment. Our ultrastructural observation of DNA traps in intimate contact with a single tumor cell exhibiting early cytopathic alterations is consistent with these experimental data. Thus, in this example of advanced gastric carcinoma with a high density of ETotic eosinophils, cytotoxic cationic proteins, contained in FEGs, and extracellular DNA traps may cooperate synergistically to create an antitumor microenvironment.

Given that ETosis was identified in only one gastric cancer case within our small series, establishing a significant association between ETosis occurrence and clinical characteristics is not feasible in the present investigation. However, our results underscore the existence of eosinophil ETosis in the tumor microenvironment, which implies a potential role in gastric cancer. Further studies on larger patient cohorts will be necessary to investigate its clinical significance and potential prognostic implications.

## Abbreviations

ETosis: extracellular trap cell death
FEGs: free extracellular granules
TATE: tumor-associated tissue eosinophilia

## Declarations

### Availability of data and materials

All relevant data are contained within the manuscript.

### Copyright

© The Author(s) 2025.
